# Does EMDR Therapy Have an Effect on Memories of Emotional Abuse, Neglect and Other Types of Adverse Events in Patients with a Personality Disorder? Preliminary Data

**DOI:** 10.3390/jcm10194333

**Published:** 2021-09-23

**Authors:** Laurian Hafkemeijer, Annemieke Starrenburg, Job van der Palen, Karin Slotema, Ad de Jongh

**Affiliations:** 1 GGZ Delfland, Sint Jorisweg 2, 2612 GA Delft, The Netherlands; a.starrenburg@ggz-delfland.nl; 2 Department of Research Methodology, Measurement and Data Analysis, University of Twente, 7522 NB Enschede, The Netherlands; j.vanderpalen@mst.nl; 3 Department of Epidemiology, Medisch Spectrum Twente, 7522 NB Enschede, The Netherlands; 4 Department of Personality Disorders, Parnassia Psychiatric Institute, 2512 VA The Hague, The Netherlands; c.slotema@psyq.nl; 5 Department of Psychology, Education & Child Studies, Erasmus School of Social and Behavioral Sciences, Erasmus University Rotterdam, 3062 PA Rotterdam, The Netherlands; 6 Academic Centre for Dentistry Amsterdam (ACTA), University of Amsterdam and VU University, 1012 WX Amsterdam, The Netherlands; a.d.jongh@acta.nl; 7 Research Department, PSYTREC, 3723 MB Bilthoven, The Netherlands; 8 School of Health Sciences, Salford University, Manchester M13 9NQ, UK; 9 Institute of Health and Society, University of Worcester, Worcester WR2 6AJ, UK; 10 School of Psychology, Queen’s University, BT9 5BN Belfast, Ireland

**Keywords:** personality disorder, EMDR, neglect, abuse, adverse events

## Abstract

Background: Little is known about the effectiveness of trauma-focused therapies for memories of events not meeting the A-criterion of post-traumatic stress disorder (PTSD). Objective: Determining the effect of EMDR therapy on memories of emotional abuse, neglect and other types of adverse events in patients with a personality disorder (PD). Method: We conducted a secondary analysis of the data from our study, which aimed to determine the effectiveness of five sessions of EMDR therapy in 49 patients with a PD. Patients were divided into three different groups depending on their most prevalent type of adverse event. Data were analyzed with Generalized Estimating Equations. Results: Of all patients, 49% reported emotional neglect, 22.4% emotional abuse and 26.5% other types. Only one patient reported memories that predominantly fulfilled the A-criterion of PTSD. After five sessions of EMDR therapy, medium to large treatment effects for memories related to neglect (ds between 0.52 and 0.79), medium treatment effects for memories involving emotional abuse (ds between 0.18 and 0.59) and other types of adverse events were found (ds between 0.18 and 0.53). No significant differences in symptom reduction associated with the application of EMDR therapy among memories involving these three different types of adverse events could be revealed. Conclusions: The results support the notion that EMDR therapy is not only an effective therapy for memories related to A-criteria-worthy events, but that it also has a symptom-reducing effect on memories involving other types of adverse events. This suggests that EMDR might be a valuable addition to the treatment of PD without PTSD.

## 1. Introduction

Prevalence rates for psychological trauma among patients with personality disorders (PDs) are high [1]. A history of childhood maltreatment is disproportionately prevalent [2,3]. Development of PDs has been found to be associated with a history of various types of (childhood) adverse events, such as physical and sexual violence [4], emotional abuse [5] and emotional and physical neglect [3]. Furthermore, there is evidence to suggest that patients who experienced adverse events in their childhood are at risk of developing difficulties in attachment, which is an important psychological determinant of PDs [6,7,8]. In addition, exposure to multiple forms of childhood abuse, such as emotional abuse, has been found to be associated with the development of inadequate emotion regulation skills, including rumination and a lack of emotional acceptance [5], which are also considered risk factors for the development and maintaining of PDs [9].

Given the relatively high level of exposure to adverse childhood events in patients with PDs, it could be hypothesized that treating and processing the memories of these events will decrease symptoms and symptom clusters typically associated with PDs, such as problems with regulation of emotions, low self-esteem, social withdrawal, avoidance and mistrust. Indeed, several studies showed that trauma-focused therapy was associated with an improvement of symptoms of comorbid PDs in patients fulfilling the diagnostic criteria of PTSD [10,11,12]. However, many individuals with a PD do not meet the diagnostic criteria for PTSD because their memories do not meet the A-criterion of the DSM-5 PTSD classification [13] that defines the index trauma as ‘exposure to actual or threatened death, serious injury or sexual violence’.

Although trauma-focused therapy has been found to be effective for patients reporting memories that are typically related to the A-criterion of PTSD (such as physical and sexual violence), it is not logical per se that other types of memories respond in a similar way. For example, a study focusing on memories associated with individuals’ low self-esteem in patients with an anxiety disorder (without PTSD) showed that competitive memory training [14] was superior to EMDR therapy. The authors suggested that the memories may not be sufficiently emotionally charged for EMDR therapy to be effective: ‘Alternatively, perhaps negative self-beliefs are rooted in cognitive belief systems that are less characterized by emotionally charged and fearful memory representations and, are as a consequence less sensitive to EMDR’ [14]. Indeed, studies on emotionally neutral memories suggest that EMDR therapy is less effective when memories are less emotionally charged [15,16].

Since adverse memories involving emotional abuse and neglect are highly prevalent in patients with a PD (with 73% reporting abuse and 82% reporting neglect) [17], it is important to explore the effects of EMDR therapy on these different memory types [18,19]. The results of a randomized controlled study of patients with a PD who did not fulfil the diagnostic criteria of PTSD showed that resolving memories of adverse childhood events using EMDR therapy led to a significant overall reduction in symptoms [18]. Because the type of index memories experienced by patients involved not only physical and sexual abuse but also emotional neglect and emotional abuse, the purpose of the current study was to determine the differential effect of EMDR therapy on these memory types. We hypothesized that patients would experience significantly less psychological symptoms, psychological distress and personality dysfunctioning after the application of EMDR therapy for all types of memories. In contrast, we expected no difference in symptom reduction after TAU between the types of memories.

## 2. Methods

### 2.1. Participants

The present study is based on the data of a randomized controlled trial (NL7470) in which the beneficial effects of EMDR therapy in patients with a PD without PTSD were investigated (https://www.trialregister.nl/trial/7470, accessed on 9 January 2019) [18]. All procedures involving the trial were approved by The Medical Ethics Committee, Southwest Holland, registered as NL61845.098.17. Patients from three outpatient clinics of a specialized psychiatric institute in the Netherlands, GGZ Delfland (locations Delft, Naaldwijk and Ypenburg, The Netherlands), participated in the study. All subjects provided written informed consent. In total, a sample of 97 patients with a PD was included. Patients were randomly allocated to either EMDR therapy (*n* = 51) or a waiting list control condition (*n* = 46). After five weeks of EMDR therapy or five weeks of waiting list, patients in both conditions received TAU for their PD. Patients could participate in the study if a PD as a primary diagnosis could be diagnosed according to the Diagnostic and Statistical Manual of mental disorders (DSM-5) criteria, and if they were aged between 18 and 65 years old. A good command of the Dutch language was needed. Patients with a PTSD were excluded from the study. Other exclusion criteria were a high suicide risk (defined as having attempted suicide within the past 6 months or current suicidal intention) and severe self-harm.

### 2.2. Assessment

#### 2.2.1. General Assessment

To classify a PD, the Structured Clinical Interview DSM-5 [20] was administered. The MINI International Neuropsychiatric Interview [21] was used to rule out patients with PTSD.

#### 2.2.2. Memories of Adverse Events

The order of the memories for the EMDR therapy [22] was structured in a case conceptualization. In the first session, targets were selected based upon the patient’s current symptoms and memories of worsening or etiological adverse events specified in a timeline. For the purpose of this study, the most prevalent type of adverse event per patient was identified. Then, the memories of the patients were divided into three different groups, i.e., those involving: (1) emotional abuse (i.e., psychological maltreatment and non-physical aggression), (2) emotional neglect (emotional deprivation or the absence of a nurturing emotional environment) and (3) other types of adverse events. Only one patient experienced adverse events predominantly meeting the A-criterion. For this reason, this category was not included in the analyses.

### 2.3. Outcome Measures

#### 2.3.1. Psychological Symptoms

Measurements took place after randomization at baseline, after five weeks and at a follow-up after three months. The Brief Symptom Inventory (BSI) [23] was used to measure the severity of psychological symptoms. The BSI consists of 53 items and has good psychometric properties [24]. This self-report questionnaire assesses the overall psychological distress level, the intensity of symptoms and the number of self-reported symptoms.

#### 2.3.2. Psychological Functioning

The Outcome Questionnaire-45 (OQ-45) [25] determines the level of psychological functioning. Three domains of functioning were reported by patients: symptom distress, interpersonal relations and social role performance. This self-report questionnaire of 45 items has good psychometric properties [26].

#### 2.3.3. Personality Dysfunctioning

During the trial, it was decided that the measurement of the global level of personality dysfunctioning would be added. As a result, a subsample of 28 patients in the EMDR therapy group completed the General Assessment of Personality Disorder (GAPD) [27,28]. This self-report questionnaire evaluates two major components of disordered personality (self or identity problems and interpersonal dysfunction). High scores reflect a high level of personality dysfunctioning.

### 2.4. Treatment and Treatment Training

EMDR therapy is a trauma-focused therapy. While performing a concurrent (dual-attention) task, patients have to simultaneously apply their attention to a disturbing memory (EMDR therapy is described at: https://www.emdria.org/about-emdr-therapy/, accessed on 2 March 2021). In this study, the standard EMDR therapy protocol [29,30] was used consisting of eight phases. An average of five memories of adverse events (targets) per person were treated during the trial. After processing one memory (i.e., Subjective Unit of Discomfort = 0; Validity of Cognition = 7), EMDR therapy continued by targeting the memory of the next adverse event on the timeline.

All therapists were trained and experienced in conducting EMDR therapy. They had completed at least an EMDR Europe-accredited training of the Dutch EMDR Association. During the study, all therapists participated in supervision sessions in a small group led by an EMDR Europe-accredited trainer (AdJ) in which adherence to the EMDR protocol was verified. 

## 3. Statistical Analysis

Data were analyzed using the Generalized Estimating Equations (GEE) approach [31]; these data included patients in the EMDR group. In total, 49 patients were included in the analysis. All analyses were based on the intention to treat. A GEE approach was used to deal with the repeated measurements. GEE allows adjustment for correlations between observations. In this study, the working correlation matrix was specified as ‘exchangeable’. The mean difference per memory type was divided by the pooled standard deviation to calculate the effect sizes. Cohen’s *d* was used to assess the magnitude of effect. An effect size of ≤2 was considered as a small effect, 0.5 as a medium effect and ≥0.8 as a large effect. The differences between the three groups divided according to memory type were analyzed with chi-square tests. 

The main parameter of interest was the difference in symptom reduction after the EMDR sessions between the baseline measurement and the post-treatment measurement for the most prevalent memory type on the outcome measures psychological functioning (measured with the OQ-45) and psychological symptoms (measured with the BSI). The GAPD was completed using a subsample (*n* = 28) of the patients in the EMDR group to measure personality dysfunctioning. The second parameter of interest was the difference in symptom reduction after three months of TAU per memory type. *p*-values of <0.05 were considered statistically significant. Data analysis was performed with SPSS version 27.

## 4. Results

### 4.1. Sample Characteristics and Types of Memories

**Figure 1:**
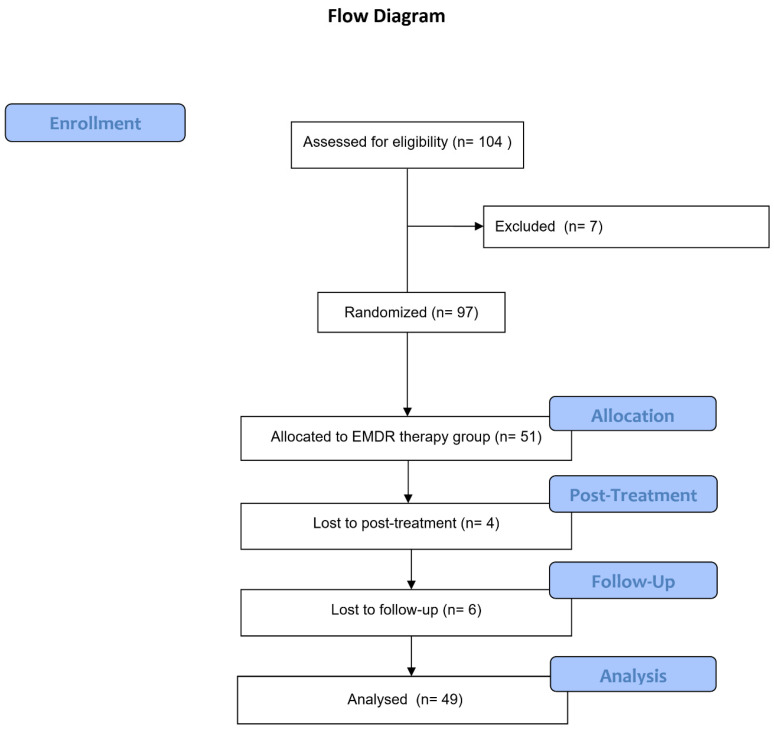
Participant flow. EMDR = eye movement desensitization and reprocessing therapy; SUD = subjective unit of discomfort; WL = waiting list; OQ = outcome questionnaire; BSI = brief symptom inventory.

In Figure 1, the flow chart of participants through the trial is shown, including the dropout and loss to follow-up. Only one patient reported memories that predominantly fulfilled the A-criterion of PTSD and was not included in the analysis.

In total, 49 patients were included in the analysis. For one patient, no target selection could be carried out because she discontinued treatment due to an occupational reason. For two patients, target selection took place, but they did not receive EMDR therapy because they lacked sufficient time. One patient withdrew from therapy after session #2 because she experienced no distress related to the chosen memories. One patient stopped after session #3 for unknown reasons. Table 1 shows an overview of the sample characteristics of the EMDR therapy group only and the prevalence of the different memory types.

Chi-square analyses showed that PD type (*p* = 0.851), age (*p* = 0.497) and gender (*p* = 0.247) were not associated with the type of adverse memory. Of all 49 patients, 49% reported emotional neglect, 22.4% emotional abuse and 26.5% reported other types of memories of adverse events as the most prevalent type of trauma. Table 2 presents the descriptive statistics of the GEE for the three types of memories.

**Figure 2:**
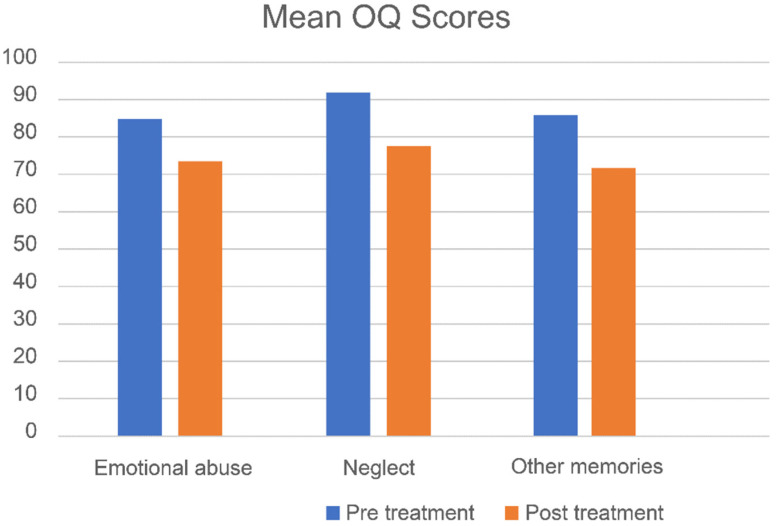
Mean OQ scores (pre- and post-treatment) for the three types of memories of patients receiving EMDR.

**Figure 3:**
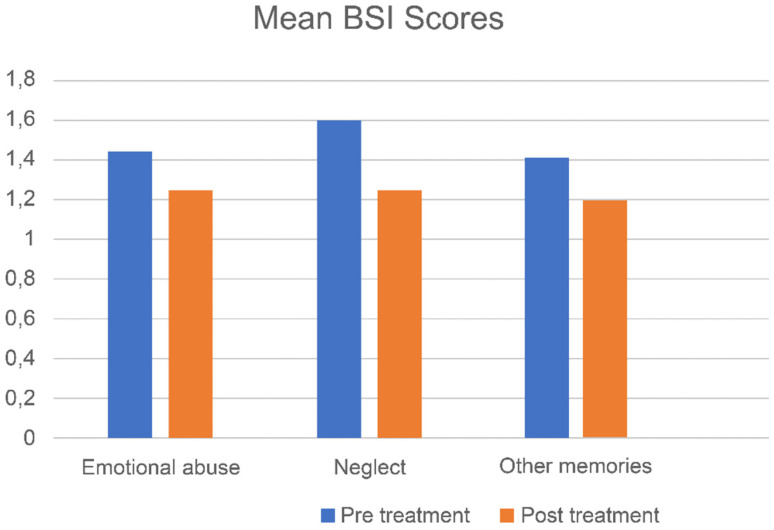
Mean BSI scores (pre- and post-treatment) for the three types of memories of patients receiving EMDR.

**Figure 4:**
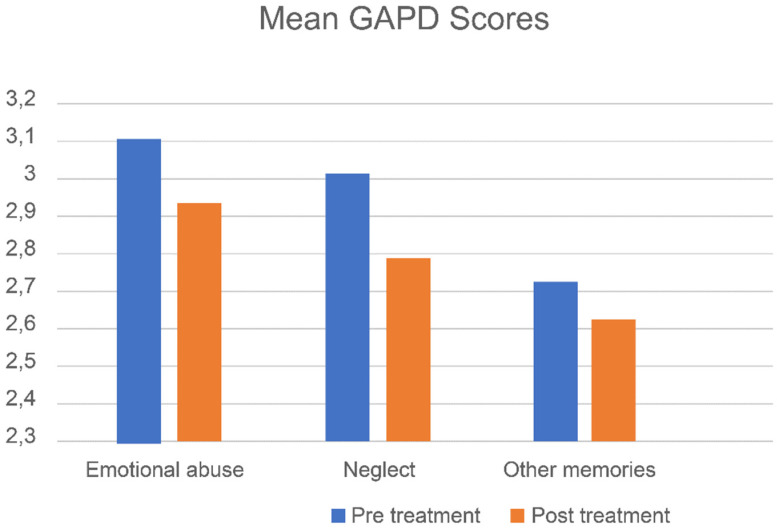
Mean GAPD scores (pre- and post-treatment) for the three types of memories of a subsample of patients receiving EMDR.

Figure 2, Figure 3 and Figure 4 show mean pre- and post-treatment scores for the three types of memories of patients that received EMDR treatment.

### 4.2. Effects of Treatment

#### 4.2.1. Overall Treatment Effects

The overall effect on symptoms related to patients’ PD has already been demonstrated in our previous publication [18]. After five sessions of EMDR therapy, participants reported significant pre-to-post reductions in psychological symptoms (*d* = 0.42), psychological dysfunctioning (*d* = 0.69) and personality dysfunctioning (*d* = 0.41). The present GEE analysis confirmed these findings of our main analysis [14], showing significant reductions in psychological symptoms (*p* < 0.001), psychological dysfunctioning (*p* < 0.001) and personality dysfunctioning (*p* < 0.014; see Table 2). 

#### 4.2.2. Memories of Emotional Abuse

Regarding memories of emotional abuse, significant pre- to post-treatment effects were found. Small to medium treatment effects were found for psychological functioning (*d* = 0.59, CI; −0.28, 1.42), and small treatment effects were observed for psychological symptoms (*d* = 0.29, CI; −0.56, 1.12) and personality dysfunctioning (*d* = 0.18, CI; −1.09, 1.43; see Table 2).

#### 4.2.3. Memories of Emotional Neglect

After the five sessions of EMDR therapy for memories of neglect, significant pre- to post-treatment effects were found with medium to large treatment effects for psychological functioning (*d* = 0.78, CI; 0.18, 1.35), psychological symptoms (*d* = 0.52, CI; −0.06, 1.09) and personality dysfunctioning (*d* = 0.79, CI; −0.83, 2.17; see Table 2).

#### 4.2.4. Other Adverse Memories

For memories other than those related to emotional abuse or neglect, significant pre-to-post reductions were found with small to medium treatment effects for psychological functioning (*d* = 0.53, CI; −0.26, 1.30), psychological symptoms (*d* = 0.31, CI; −0.47, 1.07) and personality dysfunctioning (*d* = 0.18, CI; −1.23, 1.56; see Table 2).

#### 4.2.5. Differences among the Three Memory Types

No significant differences in symptom reduction, including psychological functioning, psychological symptoms and personality dysfunctioning, were found between the three types of memories. Table 3 presents the relevant parameter estimates of the GEE analysis.

#### 4.2.6. Results at 3-Month Follow-Up

Table 2 also presents the descriptive statistics of the GEE for the three categories of memories and their effect sizes at the 3-month follow-up compared to post-treatment. By the 3-month follow-up, patients had received three months of TAU for their PD (i.e., schema-focused therapy, emotion regulation therapy and competitive memory training for PD). Small treatment effects were found for memories related to emotional neglect for psychological functioning (*d* = 0.08, CI; −0.49, 0.64), psychological symptoms (*d* = 0.05, CI; −0.51, 0.62) and personality dysfunctioning (*d* = −0.29, CI; −1.51, 0.98). Regarding memories of emotional abuse, medium treatment effects were found for psychological functioning (*d* = 0.40, CI; −0.46, 1.23), psychological symptoms (*d* = 0.46, CI; −0.41, 1.29) and personality dysfunctioning (*d* = 0.32, CI; −0.43, 1.06). For memories involving other types of adverse events, small to medium treatment effects were found for psychological functioning (*d* = 0.18, CI; −0.60, 0.94), psychological symptoms (*d* = 0.33, CI; −0.46, 1.09) and personality dysfunctioning (*d* = 0.20, CI; −0.79, 1.17).

## 5. Discussion

Perhaps one of the groups least represented in therapy outcome research is that belonging to adult survivors of emotional abuse and emotional neglect in childhood. The results of the present study show that a brief track consisting of five sessions of EMDR therapy focused on memories related to emotional abuse and other adverse events yielded small to medium effect sizes, whereas those involving emotional neglect yielded medium to large effect sizes. No significant differences in symptom reduction regarding the processing of these different types of memories could be detected.

Although, in line with our hypothesis, no differences regarding the effects of EMDR therapy among memories of different types of adverse events were found, after three months of TAU, effect sizes of evidence-based TAU (preceded by EMDR therapy) were modest. Most evidence-based psychotherapies for PDs have a long duration, and it is widely believed that more sessions are required [32,33], which may be a reason why patients improve later during TAU—an effect that was not yet measurable at the 3-month follow-up. Moreover, EMDR therapy already led to an improvement in symptoms and functioning, making it possibly harder to detect a larger difference at the follow-up. In this study, the treatment integrity of TAU was not measured and TAU was not standardized, which could also have influenced treatment outcome.

Our findings are in line with research focusing on the effectiveness of EMDR therapy in different patient groups both with [12,34] and without PTSD [35,36]. Regarding the latter, a study that investigated the effectiveness of 6–8 sessions of EMDR therapy among 26 patients diagnosed with a depressive disorder, whose memories involved non-A-criterion traumatic events (i.e., parental neglect, loss, and broken relationships), led to a significant reduction in symptoms of depression and PTSD [36]. The present results are also in accordance with a study showing beneficial effects of EMDR therapy in individuals with memories involving both typical A-criterion-worthy and non-A-criterion-worthy events (i.e., social rejection, being bullied, rejection, divorce, loss of job, humiliation or a painful incident at the dentist) [37]. Half of the 64 patients were diagnosed with PTSD, whereas the other half met the diagnostic criteria of another mental health condition. The application of EMDR therapy was associated with significant reductions in emotionality and vividness of memories, with no differences between patients experiencing memories of typical A-criterion-worthy events and memories of non-A-criterion-worthy events [37]. Hence, these results support the notion that EMDR therapy is an interesting option for resolving unprocessed memories for those pertaining to other mental conditions, such as PD.

How should we interpret the remarkable finding in this study that patients who reported memories of events that meet the A-criterion of PTSD were almost completely absent in our sample? There is a wide array of literature showing that people who have been exposed to non-traumatic events, i.e., distressing life events that do not meet the A-criterion, report levels of PTSD symptoms comparable to people exposed to events meeting the A-criterion [38,39,40]. Considerably less information in the literature is available about the opposite research question, namely, how likely it is that individuals not meeting the diagnostic criteria of PTSD will report memories of events that meet the A-criterion of PTSD. It is conceivable that if one excludes individuals with PTSD, one also excludes people who report memories of A-criteria events. Another interpretation of the finding that, in our sample of 49 patients, only one single person reported memories predominantly meeting the A-criterion is that people without PTSD are less likely to report A-criteria-worthy events, for example, due to the simple fact that they do not suffer from the consequences of it (e.g., they do not experience flashbacks or avoidance behavior). An alternative explanation for the under-representation of A-criterion memories in our sample is that the psychologists who referred patients to the study, and knew that PTSD was an exclusion criterion, unconsciously and undesirably excluded the patients who reported A-criteria-worthy events. In future research, it would be interesting to measure severity of PTSD symptoms.

Several limitations of this study should be addressed. Firstly, because this is a sub-analysis of a treatment–outcome study [18], we did not conduct a power analysis. Therefore, it cannot be ruled out that a lack of statistical power explains why we were unable to detect significant differences among memory types. Furthermore, it should be noted that due to the relatively small sample sizes, the confidence intervals of the effect-sizes for the different memory types are wide. This suggests that there is considerable uncertainty about the magnitude of the effects. Nevertheless, all point estimates are in the positive direction, lending support for a change over time, as demonstrated by the GEE analyses. Moreover, for ethical reasons, traumatic memories of patients in the control group were not inventoried, which made a comparison with the control group impossible. Secondly, we categorized patients based upon their most prominent type of memory, which might have caused a bias in the memory selection. No objective questionnaire for inventorying the memories was used. Thirdly, as indicated before, no comparison could be made between memories of events fulfilling the A-criterion of PTSD and those that did not. Accordingly, in future research, it would be interesting to compare the effectiveness of EMDR therapy in relation to memories involving events meeting the A-criterion of PTSD and those that do not. Among the strengths of the current study, we consider the inclusion of a wide diversity of memories related to adverse events, patients from three different outpatient clinics and a wide variety of PDs to have enhanced the external validity of the present results. More than 70% of the patients included in this study had been diagnosed as having two or more secondary mental health conditions, making it a representative example of clinical practice in specialist health care. This is also the first study that has evaluated EMDR therapy as a stand-alone treatment after five sessions.

## 6. Conclusions

The results provide further support for the notion that EMDR therapy is not only an effective therapy for memories related to A-criteria-worthy events, but that it also has a symptom-reducing effect on memories involving emotional abuse, emotional neglect and other types of adverse events. This suggests that EMDR might be a valuable addition to the treatment of patients with a PD without PTSD.

## Figures and Tables

**Table 1:** Characteristics of patients receiving EMDR (*n* = 49).

Variable	EMDR Group
Mean age (years)	33.9 (range 18–64)
Gender	
Male	17 (33.3%)
Female	32 (66.7%)
Personality cluster	
B	13 (29.4%)
C	20 (32.2%)
OS	16 (31.4%)
DSM-5 personality classification	
Borderline PD	12 (25.5%)
Avoidant PD	12 (25.5%)
Histrionic PD	-
Narcistic PD	-
PD OS	19 (37.3%)
Obsessive compulsive PD	6 (11.8%)
Dependent PD	-
Types of memories	
Emotional abuse	11 (22.4%)
Neglect	24 (49%)
Other memories	13 (26.5%)

**Table 2:** Descriptive statistics of the GEE of patients receiving EMDR and the three separate memory types.

					Effect Size d at Post-Treatment		Effect Size d from PT to 3 Months FU		
	*n*	BL Score Mean (SD)	PT Score Mean (SD)	3-Month Follow-Up Mean (SD)	*d*	CI Interval	*d*	CI Interval	*p*-Value (GEE) of the Difference over Time
Total sample									
OQ-45	49	87.15 (21.11)	74.35 (23.86)	69.07 (27.60)	0.57	0.16–0.97	0.20	−0.19–0.60	<0.001
BSI	49	1.48 (0.74)	1.23 (0.67)	1.06 (0.68)	0.35	−0.05–0.75	0.25	−0.15–0.64	<0.001
Type of memory									
OQ-45									
Neglect	24	91.61 (17.68)	77.66 (18.26)	75.99 (24.24)	0.78	0.18–1.35	0.08	−0.49–0.64	<0.001
Emotional abuse	11	84.55 (20.39)	73.27 (17.54)	63.78 (28.27)	0.59	−0.28–1.42	0.40	−0.46–1.23	0.005
Other	13	85.31 (20.09)	72.12 (28.55)	67.43 (23.59)	0.53	−0.26–1.30	0.18	−0.60–0.94	<0.001
BSI									
Neglect	24	1.58 (0.66)	1.25 (0.66)	1.22 (0.76)	0.52	−0.06–1.09	0.05	−0.51–0.62	0.018
Emotional abuse	11	1.44 (0.71)	1.25 (0.57)	0.99 (0.59)	0.29	−0.56–1.12	0.46	−0.41–1.29	<0.001
Other	13	1.41 (0.68)	1.20 (0.71)	0.98 (0.63)	0.31	−0.47–1.07	0.33	−0.46–1.09	0.002

BSI = brief symptom inventory; OQ-45 = outcome questionnaire 45; BL = baseline; PT = post-treatment.

**Table 3:** Parameter estimates of the GEE analysis with reference category Other Targets.

	Fixed Effects OQ-45					Fixed Effects BSI				
	Est/Beta	SE	95% CI	Wald Chi-Square	*p*	Est/Beta	SE	95% CI	Wald Chi-Square	*p*
Intercept	85.31	5.57	74.39–96.23	234.32	<0.001	1.4	0.19	1.04–1.79	1.78	<0.001
Neglect	6.3	6.64	−6.71–19.31	0.90	0.343	0.17	0.23	−0.29–0.63	0.53	0.465
EA	0.76	8.30	−17.03–15.50	0.08	0.927	0.03	0.29	−0.53–0.59	0.01	0.922
Post-treatment	−13.19	4.50	−22.00–4.38	8.60	0.003	−0.22	0.10	−0.42–0.01	4.29	0.038
Neglect Post-treatment	−0.775	5.18	−10.90–9.40	0.02	0.884	−0.12	0.16	−0.42–0.19	0.55	0.458
EA Post-treatment	1.92	6.06	−9.96–13.79	0.10	0.752	0.03	0.14	−0.24–0.29	0.04	0.852
Follow-up	−17.88	4.46	26.62–9.13	16.05	0.000	−0.43	0.13	−0.69–−0.18	10.93	0.001
Neglect Follow-up	2.26	6.31	−10.11–14.64	0.13	0.720	0.07	0.20	−0.32–0.46	0.11	0.738
EA Follow-up	2.89	7.97	−18.51–12.72	0.13	0.716	−0.02	0.18	−0.37–0.32	0.02	0.901
	Model Fit					Model Fit				
QIC	60,424.259					72.171				

BSI = brief symptom inventory; OQ-45 = outcome questionnaire 45; EA = emotional abuse.

## Data Availability

No new data were created or analyzed in this study. Data sharing is not applicable.
